# Implementation of an ergonomics intervention in a Swedish flight baggage handling company—A process evaluation

**DOI:** 10.1371/journal.pone.0191760

**Published:** 2018-03-07

**Authors:** Eva L. Bergsten, Svend Erik Mathiassen, Johan Larsson, Lydia Kwak

**Affiliations:** 1 Centre for Musculoskeletal Research, Department of Occupational and Public Health Sciences, University of Gävle, Gävle, Sweden; 2 Occupational and Environmental Medicine, Department of Medical Sciences, Uppsala University, Uppsala, Sweden; 3 Unit of Intervention and Implementation Research, Institute of Environmental Medicine, Karolinska Institutet, Stockholm, Sweden; University of Illinois at Urbana-Champaign, UNITED STATES

## Abstract

**Objective:**

To conduct a process evaluation of the implementation of an ergonomics training program aimed at increasing the use of loading assist devices in flight baggage handling.

**Methods:**

Feasibility related to the process items recruitment, reach, context, dose delivered (training time and content); dose received (participants’ engagement); satisfaction with training; intermediate outcomes (skills, confidence and behaviors); and barriers and facilitators of the training intervention were assessed by qualitative and quantitative methods.

**Results:**

Implementation proved successful regarding dose delivered, dose received and satisfaction. Confidence among participants in the training program in using and talking about devices, observed use of devices among colleagues, and internal feedback on work behavior increased significantly (p<0.01). Main facilitators were self-efficacy, motivation, and perceived utility of training among the trainees. Barriers included lack of peer support, opportunities to observe and practice behaviors, and follow-up activities; as well as staff reduction and job insecurity.

**Conclusions:**

In identifying important barriers and facilitators for a successful outcome, this study can help supporting the effectiveness of future interventions. Our results suggest that barriers caused by organizational changes may likely be alleviated by recruiting motivated trainees and securing strong organizational support for the implementation.

## Introduction

Flight baggage handlers sort, load, and unload baggage and cargo on and off transportation baggage carts, unload and load these carts for departing and arriving aircrafts, and stow baggage in the aircraft baggage compartment. Other tasks related to the arrival and departure of aircraft include towing aircraft to and from gates, attaching auxiliary power cables, and moving pylons, brake bumpers and stairs into place. Flight baggage handling occurs world-wide, and since ergonomics conditions are, to a considerable extent, determined by the transported baggage and the construction of aircraft, physical workloads appear similar for baggage handlers in all major airports. Baggage handlers show a high prevalence of work-related musculoskeletal disorders (MSDs); in a recent study, we found the one-year prevalence of low back and shoulder pain among Swedish baggage handlers to be 70% and 60%, respectively [1].

In order to reduce MSDs among flight baggage handlers in Sweden, the Vocational Training and Working Environment Council (TYA)–a council formed by employers and unions in the Swedish transport sector–initiated a project to document the prevalence of MSDs among baggage handlers, register biomechanical and psychosocial exposures in the job, and develop viable suggestions on how to improve working conditions. TYA conducted the project in collaboration with a research team and fourteen baggage handling companies operating in six Swedish airports. The project revealed a high prevalence of MSD, some occurrence of heavy biomechanical exposures, and a dissatisfaction with psychosocial factors, in particular concerning leadership and influence at work [1,2]. Moreover the baggage handlers reported that available loading assist devices were not used adequately, major reasons being that the devices were too time-consuming to use and often were not functioning due to poor maintenance.

Based on these results, an ergonomics training program was initiated by the baggage handling company and TYA together, and developed by a team including representatives of TYA, the baggage handling company, and consultants in ergonomics and behavioral science. The aims of the program were to reduce and prevent MSDs by increasing the use of loading assist devices through improved work skills and confidence in use of the devices, and to promote communication between workers. Obviously, the effectiveness of any ergonomics intervention to prevent MSD depends on two components: the extent to which the intended intervention is effective in reducing MSDs, and the extent to which the change is, indeed, adopted in the organization and by the individual workers [3,4]. In the present study, we address the latter of these two prerequisites by following the implementation of the ergonomics training program and evaluating the feasibility of the training i.e. recruitment, reach, context, dose delivered (training time and content), dose received (participants engagement), and satisfaction with the delivery, content and relevance of the training. We also evaluate the process of implementing the knowledge gained during training in practice, including the barriers and facilitators that could have influenced knowledge transfer. In the literature, evidence for the effectiveness of ergonomics interventions is inconsistent [5–7]. Reviews have shown that ergonomics intervention studies often focus only on the effect outcomes, such as exposures and MSDs, that the intervention is often designed with little consideration to theories of effective change processes, and that systematic assessments of barriers and facilitators to the implementation process are rare [8–12]. To this end, Grossman & Salas have identified ten components of organisational barriers and facilitators that show a particularly strong and consistent association with transfer of knowledge [13]. Thus, many ergonomics intervention studies appear insufficient in the context of understanding factors in the implementation process that determine the extent to which the intervention will, eventually, be successful. A better understanding of the implementation process and factors influencing the process can facilitate successful implementation of interventions, and thus increase the likelihood that the intervention leads to the intended result [5]. In the present study, we therefore aimed at answering the following questions concerning the ergonomics training program: 1) How successful was the intervention in terms of recruitment, context, reach, dose delivered, dose received and satisfaction?; 2) To which extent did intermediate outcome variables (skills, confidence and behaviors) change, and did these outcome variables differ between groups of key persons (safety officer, coordinator, instructor, manager)?; 3) Which barriers and facilitators related to trainee characteristics, training design, work environment and work organization were identified during the implementation process?

We employed a mixed methods approach involving both quantitative and qualitative methods for systematically collecting and evaluating data, similar to the approach described by O'Cathain et al. [14].

## Methods

The present study addresses a part of a larger project devoted to the work environment in the flight baggage handling sector, conducted in Sweden 2010–2012 in collaboration between TYA (henceforth referred to as the expert organisation) and our research group. Six airports and fourteen handling companies initially participated in the project and results describing physical and psychosocial exposures within the occupation have been presented to the transport sector, and documented in scientific publications [1,2]. In addition, the project group suggested general recommendations for work environment improvements based on the results of the project. Since data were collected at a company level, all participating companies received their specific results. In order to support work environment improvements within the participating companies TYA invited each company to participative workshops at the workplace. One of the companies accepted this invitation, and the expert organisation arranged a workshop for two days in May and June 2014 to discuss prioritizing work environment improvements. As a result of the workshop, the parties decided that a training program would be appropriate, aiming at facilitating transfer of skill and behavior regarding the use of loading assist devices at the worksite between key persons (KP) attending the training and their colleague baggage handlers. The study was approved by the Regional Ethical Review Board in Uppsala, Sweden (Dnr. 2014/147), and all participants signed an informed consent.

### Training program

The training was carried out during May and June, 2014, and KPs were offered one day of training in each of two topics: *Ergonomics*, which dealt with when, why and how to use loading assist devices; and *Human Factors at work* (HF), which dealt with work environment rules, norms and how to communicate at work. The expert organisation engaged three expert consultants to be responsible for the two parts of the training program, with long-term experience of the specific work environment in flight baggage handling, and established contacts with the baggage handling organization. Training sessions took place at the worksite during work hours.

### Participants in the training

All participants in the training program were key persons, recruited among 93 eligible baggage handlers identified to have key roles in the company (Table 1), i.e. safety officers (SO) (n = 16), instructors teaching the use of technical devices (n = 26), coordinators allocating tasks to baggage handlers during the shift (n = 42), and managers (n = 9). Fifty key persons participated in at least one part of the program. The Ergonomics part of the program was attended to by 43 of these key persons, the Human Factors part had 35 participants, and 28 key persons participated in both parts of the program (Table 1). Six managers, six SOs and six instructors served as informants during the evaluation process, out of which three SOs and three instructors also served as observers (Table 2).

**Table 1:** Eligible key persons, and participants in the Ergonomics and Human factors parts of the training program.

Participants	Eligible	Participants, Ergonomics		Participants, Human Factors		Participants, Both parts	
	*n*	*n*	*% of eligible*	*n*	*% of eligible*	*n*	*% of eligible*
Safety officer	16	12	75	11	69	11	69
Coordinator	42	11	26	11	26	8	19
Instructor	26	13	50	9	35	7	27
Manager	9	7	77	4	44	2	22
Total	93	43	46	35	38	28	30

**Table 2:** Methods used and time for data collection, eligible informants and response rate during process evaluation.

Data Source	Time	Informants (eligible)	Respondentsn
Course evaluation Erg	At intervention	Participants Erg (n = 43)	43
Course evaluation HF	At intervention	Participants HR (n = 35)	35
Web questionnaire (FQ)	4 months follow up	All participants (n = 50)	35
Telephone interview	6 months follow up	SOs, instructors (n = 12)	12
Telephone interview	9 months follow up	Managers (n = 6)	6
Telephone interview	4 month follow up	Observers (n = 6)	6
	5 months follow up	Observers (n = 6)	6
	6 months follow up	Observers (n = 6)	4
	7 months follow up	Observers (n = 6)	3
Company Information	Continuously	OHS manager (n = 1)	1
Course data	Continuously	Consultants (n = 3)	3

### Ergonomics training

The Ergonomics training was jointly organized by two of the consultants; one specialized in ergonomics and the other in behavioural science. The training emphasized performance of work tasks with due consideration to physical and psychosocial risk factors, risk behaviours, and consequences of risk behaviours in terms of accidents and MSDs. The training comprised two hours of theoretical teaching and a two hours of practical training in the workplace.

### Human factors at work training

The HF training was administered by the third consultant, who had a background in social science and was familiar with the company and the occupation. The training focussed on communication, feedback, attitudes and complies with work place rules and norms. The training took place during five hours, including lunch and shorter breaks, on a single day, and it mixed theory with group exercises.

### Data collection

Data for the process evaluation were collected by means of an anonymous course evaluation, a four-month follow up web-based questionnaire (FQ) and telephone interviews. Intermediate outcomes were assessed in the FQ. Table 3 presents an overview of methods used to assess process items, as well as intermediate outcomes and barriers and facilitators.

**Table 3:** Components in the process evaluation and methods used to retrieve information in the present study.

Components		Explanation	Data collection method
***Process items***			
Recruitment		Procedures used to recruit participants to the training program.	Company data
Context		Organisational aspects that may influence program implementation.	Interview
Reach		Proportion of intended target participants, measured by attendance.	Company data
Dose delivered		Number of training hours and components delivered.	Course evaluation, company data
Dose received		The extent to which participants actively engaged, interacted and used materials and resources provided.	Course evaluation, company data
Satisfaction		Satisfaction with training content and how it was delivered in terms of time, relevance and usability.	Course evaluation
***Barriers & Facilitators***			
Trainee characteristics			
	Self-efficacy	Participants’ judgments about their competence to perform a task.	FQ, interview
	Motivation	Motivation to learn and transfer knowledge.	FQ, interview
	Perceived utility of training	Perception of whether training is useful and valuable.	FQ, interview
Training design			
	Behavioral modeling	Observing and practicing target behaviors.	FQ, interview
	Error management	Practicing knowledge and skills by making errors and receiving error instructions.	Interview
	Realistic training environment	Learning and practicing in the work environment.	Interview
Work environment			
	Transfer climate	Extent to which learned skills are applied and feedback is received on performance.	FQ, interview
	Support	Supervisor and peer support including communication of goals and feedback regarding desired and accepted performance.	FQ, interview
	Opportunity to perform	Opportunities to perform new skills, e.g. modifying work to allow practice.	FQ, interview
	Follow-up	Additional learning opportunities after training period.	Interview
***Intermediate effects***			
	Skill		FQ
	Confidence		FQ
	Behavior		FQ

#### Process items

The process evaluation used a mixed methods approach, which was guided by a theoretical framework by Linnan and Steckler [15]. Six process items were addressed, i.e. recruitment, context, reach, dose delivered, dose received and satisfaction (Table 3). Information regarding the process of *recruitment* of participants, as well as the organizational *context* that may have influenced implementation, was collected by the Occupational Health and Safety (OHS) responsible at the company and also by a representative from the expert organisation. *Reach* was defined as the proportion of intended participants who attended to the program and collected through company data. *Dose delivered*, i.e. the extent to which the intended training was delivered as planned, was measured in hours and components (materials and exercises used), and *dose received*, i.e. the extent to which the training was received by the target group, was assessed in terms of whether participants were actively engaged and interacted. Immediately after the Ergonomics and HF training participants received a course evaluation. In the course evaluation of the Ergonomics training participants rated the extent to which they, 1) were engaged in the training; 2) considered the knowledge to be useful; 3) would get use of this new knowledge; 4) would consistently practice their new knowledge; 5) would be able and have opportunities to transfer this new knowledge to colleagues. All items were rated on a four point scale (“not at all” to “to a very large extent”).

Participants of the HF training rated the extent to which they were engaged in the education and learned useful communication techniques, and the likelihood that they would get use of these new skills. *Satisfaction* was rated in the course evaluation using questions relating training expectations to the actual outcome of the training. Participants were asked to rate their opinions on the time allocated to training on a four point scale (“too little” to “way too much”), and on the relevance of the training contents (“not at all” to “to a very large extent”). Overall satisfaction with the training was rated on a ten point scale from 0 (extremely disappointed) to 10 (extremely satisfied) in the four-month follow up web questionnaire.

#### Measuring intermediate outcomes

Data on intermediate outcomes were collected using a web-based follow-up questionnaire four months after the training program (Table 4). Intermediate outcomes included how KPs perceived their skills, their confidence in discussing the use of assistive devices at work, how often they used devices, how often they taught colleagues to use devices, and how much feedback they gave colleagues regarding work behavior. Out of the, in total, 50 unique participants in the program, 35 filled in the web-based questionnaire, with only a few cases of missing answers to individual questions. Participants were asked to rate nine items addressing the current state on a six point scale from “do not agree” to “fully agree”. The questions were phrased as statements, for example: “I feel confident in using devices”; “I often talk to my colleagues about the use of devices”; “My colleagues often talk about the use of devices”, and the participants were asked to rate each statement both for the situation before the training, and for the situation after. All statements are listed in Table 4 in the results section.

**Table 4:** Median of ratings before and after the training among participating key persons (KPs), as well as the difference after-before (ranges in brackets) with 95% confidence intervals (CI, and statistical significance (p-value).

	KPs n	Before median†	After median†	Median diff†	CI Median diff.	p-value*
I am confident using devices	34	4 (2–6)	5 (2–6)	0 (0–4)	0–0.5	**0.004**
I am confident teaching others	34	4 (1–6)	5 (1–6)	0 (0–5)	0–0.5	0.01
I often talk about devices	34	3 (2–6)	4 (2–6)	0 (0–3)	0–0.5	**0.004**
Colleagues often talk about devices	34	3 (2–6)	4 (2–6)	0 (0–2)	0–0.5	0.011
I often use devices	34	5 (1–6)	5 (1–6)	0 (0–2)	0–0.5	0.014
Colleagues often use devices	35	3 (1–6)	4 (1–6)	0 (-1–3)	0–0.5	**0.008**
Colleagues often ask for advice	35	3 (1–6)	3 (1–6)	0 (0–2)	0–0	0.034
I often give feedback	34	3 (1–6)	4 (1–6)	0 (0–2)	0–0.5	**0.002**
Colleagues often give feedback	33	3 (1–6)	3 (1–6)	0 (0–1)	0–0	0.025
Explicit goals are set from company	35	4 (1–6)	4 (1–6)	0 (0–2)	0–0	0.034

* p-value of the test for the median difference being different from zero; p<0.01 marked in bold.

† min and max values in brackets.

#### Measuring barriers and facilitators

Barriers and facilitators were assessed using both quantitative (follow-up web questionnaire) and qualitative methods (telephone interviews) (Table 3), in an approach based on Grossman and Salas’ adaptation (15) of the transfer model of Baldwin and Ford [16], which addresses training inputs, training outputs and conditions of transfer. Three categories of barriers and facilitators were assessed (Table 3): *trainee characteristics* (self-efficacy, motivation, perceived utility of training); *training design* (behavioral modeling, error management, realistic training environment); and *work environment* (transfer climate, support, opportunity to perform, follow up). These ten components within the three categories have been shown to have a particularly strong and consistent association with knowledge transfer in organisations [13]. A detailed description of the theoretical basis for emphasizing these ten components can be found in a theoretical overview by Grossman & Salas (15; p.103-115).

The web-based follow-up questionnaire used to assess intermediate outcomes also contained statements addressing six of the ten components above, i.e. s*elf-efficacy*: “In my profession, I am confident in transferring knowledge to improve the use of devices”; “I am an important key person for improving the use of devices in my work place”; *motivation*: “Using devices effectively demands a lot of time in practice”; *utility of training*: “This training was a good way to increase the use of devices”; *behavioral modeling*: “This training improved my ability to; discuss the use of devices with my colleagues, argue to increase the motivation for using devices among colleagues, give feedback to ensure that rules and regulations are followed by colleagues”; *realistic training*: “It has been difficult to use new knowledge because not enough time was allocated during work”; *support*: “It has been difficult to use new knowledge because lack of manager support or explicit policies”.

In addition to the follow-up questionnaire, semi-structured 15–20 minute telephone interviews were conducted with 18 randomly selected KPs, using a standardized protocol focussing on all ten components of barriers and facilitators (cf. Table 3). Informants were recruited during training and none of the approached KPs declined to participate. Interviews with safety officers (n = 6) and instructors (n = 6) were conducted six months after the training, and managers (n = 6) were interviewed nine months after the training. The interviews included questions regarding the progress of the implementation process, categorized according to the process components trainee characteristics, training design and work environment (Table 3), and the answers served as a complement to information obtained in the web-based FQ. Interviews were recorded and transcribed by the researcher.

Data on barriers and facilitators were also collected by observation. Six KPs (safety officers and instructors) were asked and all six agreed to be “observers” and give monthly follow up information by phone to a member of the research team. They were asked to observe and report on organisational barriers and facilitators occurring during the implementation, such as changes or other issues related to schedules, staffing, work tasks, colleagues’ use of loading assist devices and vehicles, as well as their attitudes to work with them.

### Data analysis

Descriptive statistics were computed for reach, dose delivered, dose received and satisfaction, as well as for the questionnaire data on barriers and facilitators.

For skills, confidence and behaviour, differences were calculated between “before” and “after” ratings. These differences were statistically tested using the Wilcoxon Signed Rank test after checking the assumption of symmetric distributions. Non-parametric 95% confidence interval for the median difference between “before” and “after” was estimated using the Hodges-Lehmann procedure. Differences in ratings between groups with different KP roles were analyzed using the Kruskal-Wallis test. Statistical analyses were performed with SPSS v. 22 (SPSS Inc, Chicago, IL) and the level of significance was set at p<0.01 to compensate for multiple testing.

Telephone interviews were transcribed, not verbatim, and quotations describing the ten barriers and facilitators (Table 3) were retrieved and organized into pre-determined categories by a member of the research team (ELB). For the 18 KP interviews, questions were determined a priori while for the interviews with observers some questions related to behaviours and attitudes were informed by the FQ. A second researcher (JL) who was also familiar with the context, the training program and the implementation process evaluated the transcribed interviews, extracting significant quotations concerning barriers and facilitators. The two researchers did this analysis independently of each other and met afterwards to reach consensus about the interpretations of the major results.

## Results

### Process evaluation

#### Recruitment

For economic reasons, the company and the expert organisation decided to focus the training programs only on baggage handlers with a specific key role, i.e. KPs (n = 93, cf. Table 1). Training dates and recruitment of KPs to training was organized by the company together with the expert consultants. Initially, twelve training dates were scheduled, six for Ergonomics and six for HF training, and six to ten KPs were assigned by the company to each of the training days, as a “mandatory task” if training dates corresponded to dates of planned regular work shifts. Between five and nine KPs were assigned to each of the six scheduled Ergonomics sessions, and between six and eight KPs were supposed to attend to each of the six scheduled HF sessions. Drop-outs prior to the training sessions were replaced if possible, and in a few cases additional participants were assigned.

#### Context

According to an interview with the responsible OHS officer at the company, it was not considered practically or economically feasible to let all employees attend to the training. For this reason, the company decided to only train KPs. During the time following the training, when the desired changes in work performance were expected to occur, the management of the company made several organisational changes that may have influenced the implementation process, such as changing working teams, changing systems for allocating tasks to teams, changing work schedules, and delivering a notice about staff reductions. According to the interviews with the KPs and the reports from the observers, all these changes received great attention among the baggage handlers and created a negative working atmosphere, which obstructed attempts by KP and the OHS to emphasize the importance of training and use of loading assist devices.

#### Reach

Attendance rates showed that 43 KPs (mean age 40 years; 47% of eligible KPs) participated in the Ergonomics training and 35 KPs (mean age 43 years; 38% of eligible KPs) in the HF training and 28 KPs (31% of eligible KPs) completed both trainings (Table 1). Reasons for not attending were, for instance, a changed work schedule, sick-leave, parental leave, and acute assignments at work.

#### Dose delivered

All training sessions were delivered by the same experts and with the same content and time schedule. Overall, the sessions were delivered as originally planned.

#### Dose received

Participants of the Ergonomics training rated that they “to a large or to a very large extent” were engaged in the education (86%), considered the knowledge to be useful (81%), would get use of this new knowledge (77%), would practice the new knowledge (77%), were able to transfer the new knowledge (81%) and would get the opportunity to transfer knowledge to colleagues (60%). Participants of the HF training reported that they “to a large or to a very large extent” were engaged in the education (71%), learned how to use skills (83%) and probably would use their new skills (69%).

#### Satisfaction

According to the course evaluations, participants were more satisfied with time allocated to the HF training (80%) than to the Ergonomics training (58%). The practical and theoretical parts of the Ergonomics training were rated separately, and satisfaction was higher for the theoretical part (88%) than for the practical part (46%). Both the HF and Ergonomics training were rated to be “to a large or to a very large extent” relevant by 89% and 88% of the participants, respectively, and 91% and 88% of the participants, respectively, were satisfied with the content.

### Intermediate effects

According to the follow-up questionnaire, participants significantly (P<0.01) increased their confidence in using and talking about the use of devices and in giving feedback to colleagues (Table 4). Colleagues were reported to more often use the assistive devices after the training than before. Furthermore, all other intermediate effects were trending toward improvement and therefore provide convergent support for the four items found to be significant at the p < .01 level. Since ratings did not differ between KP groups (p>0.05 for all rated items), Table 4 shows results for all KPs independent of group.

Overall satisfaction with the training, reported four months after the program, was 7.2 (SD 1.9) on a ten-grade scale for the HF program, 7.5 (SD 1.9) for the Ergonomics theory part, and 7.2 (SD 2.0) for the Ergonomics practical part.

### Barriers and facilitators

#### Trainee characteristics

With regards to the *self-efficacy* component, half (51%) of the participants stated in the follow-up questionnaire (FQ), that they were important KPs for work behavior and the use of loading assist devices at the worksite. Only 37% agreed that an efficient use of loading assist devices required extensive time in practicing. According to the interviews, participants generally perceived that they were confident and believed in their ability to apply competences and learned skills in using devices as well as in communicating and training colleagues, although some of the managers were less certain about training colleagues.

Interviews with KPs showed that all safety officers and instructors (SOI) were *motivated* to learn by training; they strongly believed that training would lead to more efficient use of devices. However, some managers argued that training was useless and that “nagging is the only way”. Some managers reported that baggage handlers did not use ergonomics devices either because they did not realize that it was important, or because devices were broken, or because they feared that devices would break during loading and thus cause flight delays. Managers were aware that devices were often broken but had not taken action to fix this problem: “seven of our conveyor belts are out of use and then they load without instead of waiting”. SOIs reported that they would use loading assist devices themselves, but that doing so could delay the aircraft, which was a reason for other baggage handlers not to use the devices. In the FQ, participants reported a high (70%) *utility of training* to facilitate use of knowledge and skills in work performance; however, they reported a wish for more practice and an annual refresher course. In the interviews, none of the managers believed in the *utility of training* to improve performance: “the course may confirm their own knowledge but it was too theoretic”, “learning by doing is the only way”; and “in reality I think someone needs to be present in the production for lecturing and surveillance”.

In summary, according to interviews and FQ, *self-efficacy*, *motivation* and the perceived *utility of training* were considered positive among the SOI participants, indicating that the *trainee characteristics* were favorable with respect to facilitating change in performance. However, interviews with managers revealed that they did not believe in the training having the intended effect, nor did they act to facilitate the use of devices, such as expected by the SOIs.

#### Training design

According to the FQ, training improved the ability of the participants to discuss the importance of loading assist devices (52%), argue and motivate the use of devices (41%) and give feedback in order to clarify workplace regulations (54%). In the interviews, both managers and SOIs reported that *behavioral modeling*, i.e. understanding and practicing expected behaviors, was important for the transfer of skills and behavior to colleagues and, thus, facilitate change in performance. Further, the participants reported a clear understanding of the consequences of not using devices; a suggestion was to provide examples to visualize “what really happens if you get musculoskeletal disorders affecting your workability”. The training time allotted to learning to anticipate risks and practice that knowledge, i.e. *error management*, was considered insufficient and the groups too big. One manager commented that “too much time was spent on theory, and the issues on how to work and when was not thoroughly explored”. However, correct use of loading assist devices was, indeed, demonstrated, and when to use them was discussed during practice sessions. One manager was excused not to attend to that session, and when interviewed two managers could barely remember if they had attended that part of training. Two participating managers felt that too much time was spent discussing issues that were specific to one aircraft, and finding a suitable aircraft for practice; this suggests that a better planning of the practical training would secure a more efficient use of time. On-the-job training after the program, i.e. *realistic training*, was reported in the FQ as unsatisfying by 28% of the participants, answering that it was difficult to practice the new knowledge and skills due to time limits. While some of the interviewed participants stated that the opportunity to train between regular work tasks was limited, some managers claimed that workers could easily find time for training during regular work; “it’s about prioritizing”. However, two managers clearly stated that shortage of workers made training during work shifts impossible; the SOIs agreed in this opinion.

In summary, the theoretical part of the training regarding faulty performance, desired learning behavior, and ergonomics consequences of not practicing safe behavior was reported in the interviews to be satisfactory by both managers and SOIs. In contrast, opportunities to observe and practice behaviors, both during the course and in job training afterwards, were reported to be unsatisfactory and in need of reconsideration when designing future training programs.

#### Work environment

The reasons reported in the FQ for not using new knowledge and skills in daily work were lack of an explicit management policy regarding when and how to use devices (34%), and lack of manager support (26%). In the interviews, SOIs reported a lack of manager *support* for prioritizing ergonomics, and a lack of situational prompts reminding workers to use devices and skills. Further, the SOIs frequently reported insufficient communication from managers, and lack of goals concerning expected work performance. Thus, a negative *transfer climate* was repeatedly reported by SOIs as a barrier for a change in work performance. Only one SOI found that the use of loading assist devices had increased following training, and speculated that longer work shifts caused physical load to accumulate, which in turn “forced” the use of devices in order to cope with that load. In contrast to the negative perception of transfer climate reported by SOIs, managers perceived they were good at peer *support* and feedback, even if they described it to be situationally bounded. They described it to vary over time with organisational changes, like lay-offs, new work schedules and work team changes. One of the managers commented that “sometimes it is very easy to be straight forward and sometimes it is not, and you must give the workers “treats” to have them do their ordinary work. *Opportunities to perform* or apply new skills on the job were limited because regular work needed to be done concurrently, and because equipment such as rampsnakes (a lifting device on the ramp) and other loading assist devices were lacking or broken. One of the SOIs reported that insecurity could be an issue: “I think some are ashamed to practice, afraid of not doing it right”. Managers agreed that devices were old and in need of frequent maintenance, but did not spontaneously discuss and acknowledge their own legal responsibility for providing necessary devices for a safe work performance. Post-training *follow-up*, provided from the expert organisation was missing and would have been appreciated according to both SOIs and managers; one SOI commented “I do not even know from whom to expect a follow up of the training”. All parties generally agreed that training was not mentioned again after the course, and neither managers nor the expert organisation initiated or conducted any follow-up activities.

In summary, no significant change in performance following the training was reported in the interviews. The transfer climate (peer *support*, *opportunities to perform* new skills, and *follow up*) was a critical barrier for achieving the desired change in behavior.

#### Work organisation—Overall barriers and facilitators

The information collected by the observers (4–7 months after training) indicated that the work environment conditions were not optimal for successful transfer of ergonomics knowledge and skills at the worksite. Focus at that time was on organisational changes, for example, changes in work schedules, staff lay-offs, and introduction of a new working system which was tested for six weeks. This system included scheduling daily work tasks by a computerized system, which directed the individual handler to which flights to load/unload only one to two hours ahead, instead of being handed a team-based work plan for the entire shift. This new approach was reported to lead to a major change in both work schedules and teams, the latter being reduced from four to two workers. These issues were substantial barriers influencing the psychosocial work climate negatively; the baggage handlers did not believe in the effectiveness of the organisational change since it had been tested before and experienced not to be positive. Observers reported that according to the baggage handlers, these organisational changes increased work pressure and made it harder to perform the work well; logistics were more complicated, and communication was not supported. The organizational change was not appreciated by managers or the union either. Observers reported that the new organisation was associated with an increased occurrence of flight delays, and with mail and baggage not being loaded due to a shortage of employees. Also, the baggage handlers got more fatigued under the new scheduling system, and sick leave rates allegedly increased. In contrast, one observer reported an increase in use of loading assist devices and said, “we are fewer workers now and I think they perceive work is harder now and you really need to use the devices to be able to work all day. Also, there is no reason to work faster to aggregate time, since there is no time anymore for extended breaks”. Observers reported that the implementation would have benefited from managers being more visible in the production, reinforcing the importance of ergonomics by reminding baggage handlers to use the devices, and providing positive feedback. Some observers believed that their managers may have needed support from senior level management and the expert organisation to be able to provide the desired support.

In summary, as suggested by the observer reports, barriers of organisational changes, staff reduction and job insecurity created a work climate in which the conditions for engagement in training and changing work behavior as intended were not altogether favorable.

## Discussion

According to TYA and their affiliated baggage handling companies in Sweden, several interventions aiming at improved health have previously been implemented within the baggage handling occupation by employers, unions and other stakeholders. This present study gives, for the first time, a description of the implementation process, including barriers and facilitators for an effective implementation of the intended ergonomics intervention. Thus, we contribute to understanding the second of two prerequisites for any ergonomics intervention to be effective in reaching an ultimate goal of preventing or reducing MSDs, i.e. that the intervention has, indeed, a potential to influence MSD, and that it is implemented according to intentions [3].

The evaluation of the present implementation demonstrated that the training was delivered as planned (*dose delivered)* and that *dose received* and *satisfaction* were both rated high. Positive, significant changes (p<0.01) were identified after the training in the participants perceived confidence in using devices, colleagues use of devices, and feedback between colleagues'. However, according to the interviews with KPs, the training program did not lead to the desired effect of a general improvement in use of loading assist devices among the baggage handlers. Barriers were identified in all four categories: *trainee characteristics*, *training design*, *work environment* and *work organization*, and the main facilitator was found in the category *trainee characteristics*. These barriers can give a possible explanation to why the training program did not result in an improvement in the use of loading devices despite positive changes in confidence using devices and giving feedback to colleagues. The possible influence of barriers and facilitators on the use of the devices will be discussed in more detail below.

Facilitators for the transfer of training into practice observed in the present study include the safety officers and instructors. SOIs believe in training leading to improved use of loading assist devices, they were *motivated* to learn by training, and the perceived *utility of training* to facilitate use of knowledge and skills was high among all participants. According to the literature, motivation before, during and after training is a crucial prerequisite for effective transfer of trained skills to the worksite [13]. Moreover, experiencing intervention advantages during training is an important promotor of the adoption of ergonomics interventions. This was confirmed in a study investigating the use of a slide-board to transfer patients among emergency medical service professionals. The use of the slide-board device was positively predicted by *previous tool experience* and *the experience of ergonomics advantage in use* [17]. In contrast to SOIs motivation and positive attitudes towards the training observed in the present study, managers were not convinced that the use of loading assist devices would increase through more training. This lack of belief in the utility of the training may have acted as a barrier towards supporting the use of the devices among baggage handlers. The identified barriers in this study, i.e. *lack of support* and *time to practice*, were similar to those found when implementing interventions in other occupational settings, including kitchens [18] and offices [19].

The facilitators found in this study, i.e. self-efficacy, motivation and perceived utility of training, all belonged to the process evaluation component *trainee characteristics* (Table 3). This indicates that the sampling strategy to recruit company KPs was a good approach, and may have been instrumental to the positive intermediate outcomes of *confidence* and *behavior*. According to the interviews, participants generally believed in their ability to apply competences and learned skills in using devices, communicating and training colleagues. A meta-analytic review confirms that investment in training may positively affect performance, but also concludes that the factors that influence the transfer of training are not sufficiently known [20]. A study by Velada and colleagues showed higher self-efficacy to be significantly related to transfer of training over time in a large grocery organization [21]. The authors suggested that in order to enhance transfer of training the company should give trainees the ability to transfer learning, reinforce the trainee's beliefs in their ability to transfer, ensure that training content is retained over time, and provide feedback on job performance.

In this case, the training was intended to influence behavior and transfer of knowledge in the worksite but the ***training design*** of the intervention did not allow satisfactory transfer of training. The training was planned by expert consultants based on participatory meetings with employer, employee and union representatives. A participatory intervention design is generally recommended, exemplified by a study concluding that important success factors in ergonomics interventions include carrying out an initial problem identification leading to a proposal, which is then followed by direct participation of workers in designing the intervention [5,22]. A review by Montano et al. summarizes authors' explanations of why their interventions had limited success, one being that employees did not sufficiently participate in preparing the intervention, and others being a lack of communication and motivation to support the intervention and comply with organizational changes [8]. In another systematic review, van Eerd et al. emphasized the importance of having the right team members [5]. They concluded that workers, supervisors and specialists with the right skills and knowledge were key actors in implementation and that voluntary involvement of workers representing larger groups appeared to be an efficient initiative. A problem raised by the participants was that the time devoted to observing and practicing behaviors during the training program was too limited to allow sufficient *behavior modeling*. The importance of training time for behavior modeling was emphasized by a meta-analytic review [23] showing that an extended period of training leads to better skill development. To practice in realistic settings during and after training (*on the job training)* and generate scenarios to practice problem solving *(error management)* was, in the case, deemed impossible by the SOIs due to time constraints and lack of manager support. In contrast, some managers argued that the alleged time constraint was a matter of “prioritizing” and not a barrier for practice. This resembles a laissez-faire leadership style [24], which does not support training implementation and, thus, represents a substantial barrier. Reasons for this leadership behavior in the present setting were not obvious, but managers may have lacked in motivation and support for ergonomics interventions, or they may have considered workers to lack the competence to work independently, make decisions and accomplish tasks with limited guidance. Further, transfer of training has been shown to be greatest if participants are instructed to set goals on which new competences to obtain [23]; in the present intervention, goals may not have been explicitly expressed.

In the ***work environment*** category, lack of supervisory *support* influenced transfer of training negatively, and participants asked for “a more visible leadership”. Supervisory support was shown in a review as an important factor for workers well-being and a low degree of work stress [25] and a visible leadership has been identified as a key behavior promoting both productivity and good health at the organisational level [26]. In particular, safety officers in our study asked for manager support in giving feedback to workers breaking rules concerning use of loading assist devices. The relevance of this request is supported by a meta-analysis of behavior modeling training showing that training attendance and eventual behavior effects were facilitated if managers also underwent training and used rewards and sanctions in the work environment [23]. The need for managers expressing clear rules and norms is supported also by another systematic review concluding that a greater implementation involvement and support from managers is associated with a more effective intervention implementation and a greater likelihood of positive outcomes [27]. Further, studies have shown that intervention outcomes were more positive if managers were responsible for the implementation, provided that they allow employees to spend the necessary time on intervention activities [28]. The importance of manager involvement, which appeared to be insufficient in the present process, suggests that a better integration of managers is a vital prerequisite for success in future interventions in baggage handling. *Follow-up* activities were lacking, which was often pointed out during interviews with informants, reporting that neither the company nor the expert organisation following the training program showed any interest in following up the initiative. Finally, organisational restructuring and staff reduction were external events influencing the implementation, while beyond the control of the expert organization. This was also reported as an explanation to limited success of interventions in the review by Montano et al. [8].

### Strengths and limitations

The present study was based on a systematic collection of an extensive amount of information during the implementation process. Moreover, the structure of barriers and facilitators were based on a theoretical framework, the lack of which has been emphasized as an important general weakness in previous implementation research [9,29]. In observing the six process items *dose delivered*, *dose received*, *context*, *recruitment*, *reach* and *satisfaction*, this study included more than the average number of items addressed (i.e. 3.9, range 1–8) in process evaluations according to a systematic review of studies of process and effect evaluations in worksite health promotion programs [9]. In the quality assessment procedure applied by this review, the number of reported process variables was graded positive if four or more variables were evaluated. Only four of the 22 reviewed studies reported six or more items, putting this study in the top echelon among process evaluations in this respect.

Two researchers, (ELB and JL) took part in the process of analyzing the interviews. They read the texts independently and afterwards discussed their interpretation of contents and categorizations. This was performed in order to increase the precision in the analysis. Both researchers were active in the present project and thus familiar with both the context and the procedure used for data collection. We believe that this consensus approach led to clear and consistent results, and we trust that the same major findings had been identified by other pairs of researchers.

This study also has some limitations. We only studied one company and economical considerations dictated recruitment to only include employees with the specific key roles of being a safety officer, instructor, manager or coordinator, rather than recruitment of all employees. The selection of these key persons was supported by a systematic review suggesting that workers, supervisors and specialists (with regard to a mix of skills and knowledge) are key-actors in the process of participatory ergonomics interventions [5]. In the recruitment process, participants were randomly selected from the groups of KPs at the company and some may have dropped out because they were not willing to participate. The selection *per se* was reported by the participants as a limitation for the success of the implementation of the intervention. Also, the implementation could have been facilitated by a brief overview presentation to staff not attending the training program, but this was not done. *Reach* for the training program–which is a measure of recruitment effectiveness [15]–was only 47% and 38% for the Ergonomics and the HF program parts, respectively. While this may appear as a limitation, this reach is similar to that obtained in other workplace health promotion programs; typically below 50% [30]. Another study limitation was that we did not measure intermediate effects at two different time-points, so as to observe whether effects changed over time. Pre-training conditions were assessed four months after training, which suggest some risk of recall bias. The time line of the implementation process was decided by the company and the expert organization together with no influence from the research team, and due to shortage of time it was not possible to collect data prior to the implementation.

### Generalizability and implications

The present process evaluation was conducted in only one company, but since the tasks associated with baggage handling are similar in all Swedish baggage handling companies (2), we believe that this process evaluation can be useful in showing important barriers and facilitators of implementation even for interventions practiced in other companies.

Training participation (recruitment) in this study was not altogether successful according to the numbers, and may have been a major explanation to the low reach. In future interventions, we recommend another recruitment process, including approaching a broader group of potential participants. In the planning phase, the training implementation would, according to the results, benefit from a stronger support among senior, middle and first-line managers than what we observed. Further, to facilitate intended changes of behavior, the training needs to prioritize practical exercises, and include repeated follow ups of competences and behavior.

## Conclusion

In conclusion, the implementation process was successful regarding *dose delivered*, *dose received* and *satisfaction*. The training intervention did result in a better confidence among participating key persons in using devices and giving feedback to colleagues, and the key persons even observed an increased use of loading assist devices by their colleagues; these intermediate outcomes were similar in different groups of key persons. Main barriers to implementation were lack of explicit management policy and support regarding when and how to use assist devices and practice correct behaviors, lack of training follow-up, staff reduction, and job insecurity. Facilitators were a positive self-efficacy, high motivation, and perceived utility of the training among the participants in the program.

The studied intervention did not bring about the intended changes of a general improvement in the use of loading assist devices among the workers; the effectiveness of the implementation process may, thus, be questioned. Thus, the intervention would not be expected to lead to the intended reduction and prevention of musculoskeletal disorders. The comprehensive evaluation of the implementation process can help in understanding combinations of barriers and facilitators in different categories that could lead to an insufficient outcome; this information can, in turn, support a more successful implementation of future ergonomics interventions in baggage handling. In this particular case, a strong organizational support for the implementation would likely have reduced the impact of the identified barriers associated with organizational changes. Also, we find that a particularly important facilitator of implementation would be to recruit dedicated and motivated trainees for the intervention program.

## Abbreviations

MSD: Musculoskeletal disorders
TYA: Vocational Training and Working Environment Council
KP: Key-persons
HF: Human factors
SO: Safety officers
FQ: Follow up questionnaire
OHS: Occupational health and safety
SOI: Safety officers and instructors
